# Qualitative analysis and clinical implications of lipid layer thickness variability: A retrospective, cross-sectional study

**DOI:** 10.1371/journal.pone.0357020

**Published:** 2026-09-15

**Authors:** Jiyoung Emily Lee, Dong-Kyu Kim, Hye Yeon Yoon, Woong-Joo Whang, Ho Sik Hwang, Hyun-Seung Kim, Reiko Arita, Eun Chul Kim, Kyung-Sun Na

**Affiliations:** 1 Department of Ophthalmology, Bucheon St. Mary’s Hospital, College of Medicine, The Catholic University of Korea, Seoul, Korea; 2 Department of Ophthalmology, Yeouido St. Mary’s Hospital, College of Medicine, The Catholic University of Korea, Seoul, Korea; 3 Department of Ophthalmology, Seoul St. Mary’s Hospital, College of Medicine, The Catholic University of Korea, Seoul, Korea; 4 Itoh Clinic, Saitama, Japan; 5 Department of Ophthalmology, Keio University, Tokyo, Japan; The Ohio State University, UNITED STATES OF AMERICA

## Abstract

Meibomian gland (MG) dysfunction impairs the tear film lipid layer, which is critical for preventing aqueous evaporation and maintaining tear film stability. While a LipiView® II-reported mean lipid layer thickness (LLT) of ≥100 nm indicates a high- or ceiling-range device output rather than a calibrated absolute LLT, mean LLT alone may overlook clinically relevant information about the dynamic behavior of the tear film. This study aimed to evaluate whether qualitative variability in 20-s LipiView® II dynamic LLT traces is associated with MG morphology and ocular surface findings in eyes with device-reported high-range LLT values. In this retrospective, cross-sectional study, we analyzed 20-s dynamic LipiView® II recordings from eyes that were initially measured to have a mean LLT of ≥100 nm. Two masked observers classified LLT pattern variability as high or low based on predefined criteria. Clinical and demographic differences between the high- and low-variability groups were assessed statistically. Out of 85 eyes, 58 (68.2%) and 27 (31.8%) exhibited high- and low-variability, respectively. The high-variability group demonstrated markedly elevated meiboscores compared to the low-variability group, indicating a greater degree of MG dropouts (*p* = 0.042). Besides elevated meiboscores, patients in the high-variability group suffered from substantially worse dry eye disease (DED) symptoms, a trend toward higher corneal and conjunctival staining scores, and significantly increased blinking frequency (*p* < 0.05). Qualitative evaluation of LLT revealed increased meiboscores, greater severity of DED symptoms, and increased blinking frequency even in eyes with device-reported high-range mean LLT. LLT pattern analysis may warrant evaluation as an adjunctive approach for the early detection and targeted management of MG dysfunction-related evaporative DED.

## Introduction

Meibomian gland dysfunction (MGD) is a chronic, diffuse abnormality of the meibomian glands (MGs), resulting in terminal duct obstruction and/or quantitative or qualitative alterations in meibum secretion [1–3]. MGD encompasses both morphological changes in MGs and functional abnormalities in their secretions [3]. The tear film lipid layer (TFLL) plays a critical role in preventing aqueous evaporation and maintaining tear film stability [4–6]. Meibum, the lipid produced by the MG, is the main source of lipids in TFLL [4,5].

Morphological changes in the MGs, such as gland dropout or atrophy, can be visualized using non-contact infrared meibography [7]. Numerous studies have reported a positive correlation between MG loss and MGD development, as well as tear film instability [8–10]. However, some research has suggested that the functional implications of MG loss may differ depending on the location of the dropout—whether in the upper or lower eyelid, and whether medially or laterally situated [8,11]. Moreover, structural loss alone may not fully explain the effect of MG dropout on tear films and the functional impact of dry eye disease (DED).

The LipiView® II Ocular Surface Interferometer (TearScience Inc., Morrisville, NC, USA) is a widely used device that measures the lipid layer thickness (LLT) by capturing color interference patterns [12]. Within a 20-s recording, it provides both an average LLT value and a dynamic visualization of LLT fluctuations over time. Although a thicker lipid layer is generally thought to confer greater resistance to evaporation, reported LLT cutoffs associated with tear film stability vary widely across studies, ranging from approximately 60–80 nm [13,14], and a numerically high LLT does not necessarily indicate a stable tear film [15]. Moreover, interferometric color analysis has an inherently narrow unambiguous measurement range, and the LipiView® II displays values exceeding 100 nm only as “100+” rather than as exact figures, limiting the interpretability of absolute LLT values in this range. Furthermore, LLT measurements are influenced by multiple factors, including the condition of the underlying aqueous mucin layer and blinking pattern [16]. Therefore, LLT alone may not accurately reflect the functional integrity of the MGs or overall tear film stability.

During blinking, the lipid layer is redistributed across the ocular surface, with LLT demonstrating dynamic changes between the downstroke and upstroke phases [17]. Although the LipiView® II provides valuable mean LLT measurements, averaging complex tear film fluctuations into a single value may potentially overlook critical information about the tear film’s dynamic behavior [12,14,16]. Previous studies have primarily relied on average LLT measurements, which may not capture the functional nuances of the TFLL behavior during the blink cycle. Because blinking does not necessarily redistribute the TFLL uniformly, the reliance on a simple average value risks overlooking localized thinning or instability. Therefore, in eyes with a device-reported high-range mean LLT (recorded as “100+”), in which absolute values cannot be meaningfully compared, this study aimed to investigate whether the qualitative dynamic variability pattern of LLT, as visualized using LipiView® II, rather than its numerical value, is associated with established clinical indicators of MGD and ocular surface health. This study endeavored to enhance comprehension concerning tear film dynamics and improve diagnostic approaches for MGD-related DED by focusing on both the morphological and functional aspects of MGD.

## Materials and methods

### Study design and patient recruitment

This retrospective, cross-sectional study employed a census of all patients aged ≥18 years who underwent DED evaluation at Yeouido St. Mary’s Hospital from August 2021 to August 2023. This study was approved by the Institutional Review Board (IRB) of Yeouido St. Mary’s Hospital (approval no. SC25RISI0013) and conducted in accordance with the tenets of the Declaration of Helsinki. The IRB waived the need for written informed consent from the participants due to the retrospective nature of the study and the use of de-identified patient data. Data for this retrospective study were accessed for research purposes on 15 March 2025. The authors had access only to de-identified data and did not access any information that could directly identify individual participants.

Patients who visited the clinic during the study period and underwent DED evaluation were included regardless of the diagnosis. The DED evaluation included assessments such as tear break-up time (TBUT), MG structure (meibography-based meiboscore) and function (meibum quality and expressibility), mean LLT, and symptom assessments. Although the study was not limited to patients diagnosed with DED, the focus was specifically on participants with device-reported high-range mean LLT (≥100 nm or 100+). In the LipiView® II system, LLT values exceeding 100 nm are displayed as “100+” rather than providing exact numerical values.

Exclusion criteria were as follows: (1) patients aged <18 years or those with (2) a history of ocular trauma or surgery, including punctal plug insertion, within the past 3 months, (3) any disease affecting the ocular surface, such as ocular infection, allergy, or systemic autoimmune diseases, for instance Sjögren’s syndrome, (4) current contact lens use or contact lens wear within the past three months, (5) use of topical ocular medications other than artificial tears, including topical or systemic steroids, or (6) inability to complete the required questionnaires or cooperate with the examination procedures.

Demographic information and medical history, including histories of hypertension, diabetes mellitus, and dyslipidemia, were obtained during routine clinical evaluations. The ocular history obtained during routine clinical questioning was used to apply the study exclusion criteria. Standard Patient Evaluation of Eye Dryness (SPEED) questionnaire scores, which assessed subjective DED symptoms before ophthalmic examination, were also recorded. The SPEED questionnaire, developed in 2005 by TearScience (Morrisville, North Carolina, USA) and used in both clinical and non-clinical studies, is an eight-item short form that assesses symptom frequency and severity. The total scores range from 0 to 28, with higher scores indicating more severe DED symptoms [18].

### Ophthalmic examinations

#### Examination protocol.

All examinations were performed according to the standardized dry eye examination protocol of our institution, which specifies a fixed sequence with a minimum interval of 5 min between tests: LLT evaluation, TBUT measurement, corneal and conjunctival staining, MG evaluation, and non-contact meibography. Ophthalmic examinations were performed using a slit-lamp biomicroscope (Topcon SL-D701; Topcon Corp., Tokyo, Japan) by a corneal specialist, K.S.N.

#### Tear and ocular surface evaluation.

TBUT was measured using a fluorescein-based slit-lamp. A fluorescein sodium-impregnated paper strip (Haag-Streit International, Köniz, Switzerland) with a drop of topical proparacaine hydrochloride solution, Alcaine eye drops 0.5% (Alcon Ophthalmic Products Co., Ltd., Seoul, Korea), was instilled into the inferior conjunctival sac. The interval between a complete blink and the first occurrence of a visible dry spot on the tear film was measured under cobalt blue illumination using a slit-lamp biomicroscope with a yellow-barrier filter. The measurement was repeated three times, and the average value was calculated. Among all values, only the average TBUT was collected. After evaluating TBUT, corneal and conjunctival staining scores based on the Oxford grading scheme (scoring from 0 to 5 per area) were measured. The corneal staining score was recorded from 0 to 5, whereas the conjunctival staining score was calculated as the sum of the nasal and temporal bulbar conjunctival regions (each graded 0–5), yielding a total range of 0–10.

#### MG evaluation.

MG function was assessed by evaluating meibum quality (MQ) and meibum expressibility (ME) by a single investigator. The ability of the eight MGs situated in the central region of the lower eyelid to secrete meibum was evaluated following the application of mechanical pressure using a handheld Meibomian Gland Evaluator^TM^ (TearScience Inc., Morrisville, NC, USA) [19]. ME was scored on a scale of 0–8, reflecting the number of central glands capable of expressing meibum. MQ was graded on a scale from 0 to 3 for each gland according to the following criteria, which were modified from the grading system described by Mathers, et al. [20]: 0 = clear and normal meibum expressed; 1 = yellow in color, without increased viscosity; 2 = yellow in color, with increased viscosity; or 3 = toothpaste-like consistency. The scores for all eight glands were summed to yield a total MQ score ranging from 0 to 24. MG morphology was assessed using non-contact infrared meibography, a LipiView® II ocular surface interferometer, by everting the upper and lower eyelids. MG dropout scores, also referred to as meiboscores, were assigned to each eyelid based on the degree of gland dropout according to the grading system described by Arita, et al. [7]: grade 0 (no gland dropout), grade 1 (<1/3), grade 2 (1/3–2/3), and grade 3 (>2/3 gland dropout of the total meibomian area). The meiboscore was calculated by summing the scores obtained from the upper and lower eyelids.

#### LLT measurement and variability analysis.

**Fig 1:**
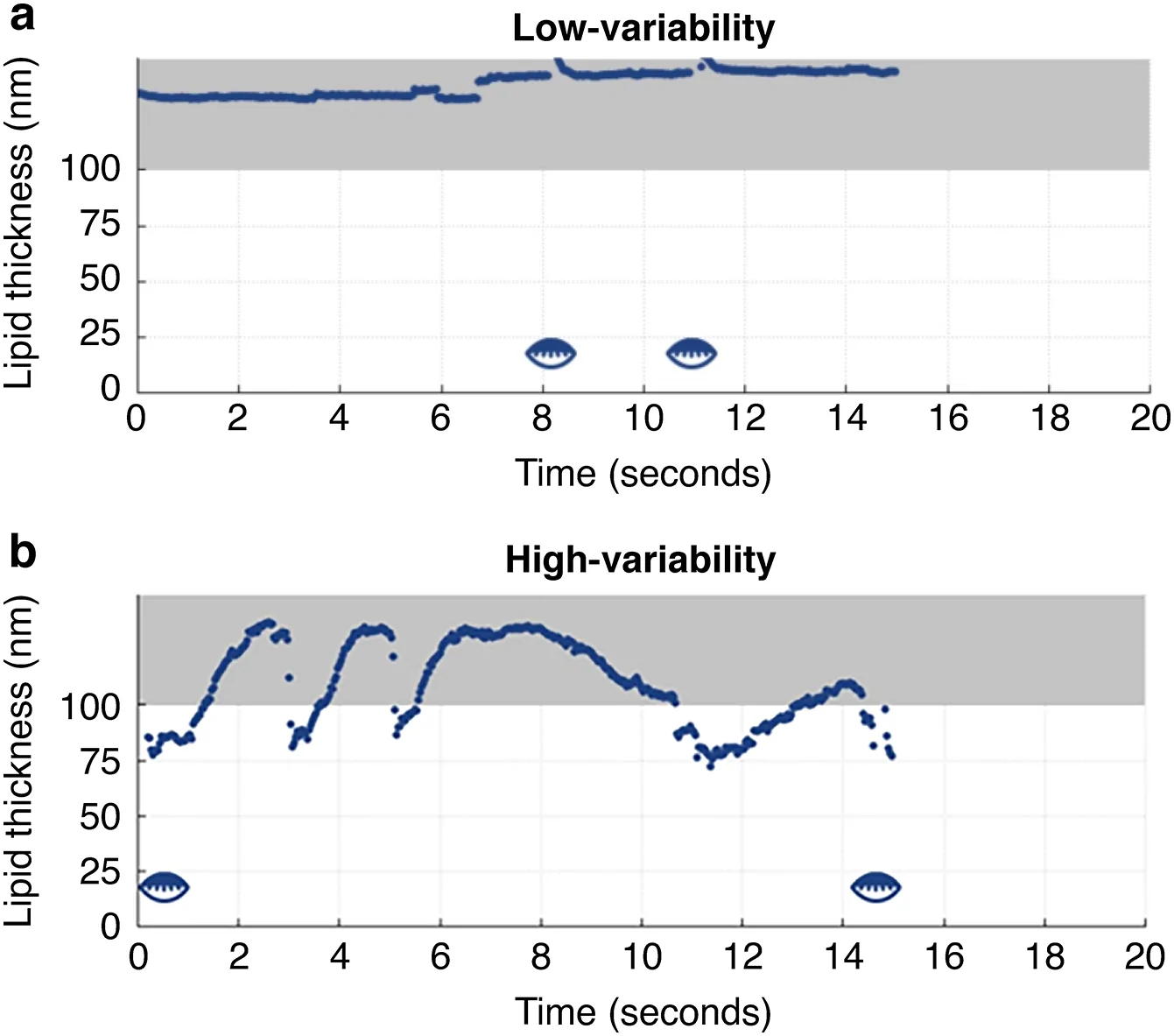
Representative lipid layer thickness variability patterns. Examples of LipiView® II dynamic lipid layer thickness (LLT) traces over a 20-s recording period, illustrating (A) a high-variability (HV) profile and (B) a low-variability (LV) profile. In the HV example, LLT fluctuations exceed a 25 nm range, SD > 10 nm, display abrupt slope transitions, and have a maximum-minimum difference >25 nm, which is consistent with the HV classification criteria. Eyes that did not fulfill any of the HV criteria were classified as having LV. Variability was assessed based on the overall shape of the LLT trace and was independent of the number of blinks. Blink events (indicated by the eyelid symbols on the time axis) occurred in both profiles; therefore, the lower variability in (B) reflects a more stable lipid layer rather than the absence of blinking.

The mean LLT was measured using a LipiView® II interferometer, which provides device-derived interferometric estimates of TFLL, reported in nanometers (nm) or interferometric color units (ICUs). The conversion from the recorded interference pattern to a thickness value relies on the manufacturer’s proprietary fitting algorithm, which is not publicly disclosed. Briefly, the patients were instructed to focus on a specialized camera that recorded a 20-s video capturing the interference pattern of the tear film. The device measures LLT within a defined region located approximately 1 mm above the inferior tear meniscus, and the reported LLT represents an average over both this region and the 20-s acquisition period [21]. This recording was subsequently analyzed to obtain measurements in ICUs, with one ICU being approximately equivalent to 1 nm of LLT [14]. In addition to the mean LLT, the device reports the minimum and maximum LLT, as well as the standard deviation (SD) of LLT over the recording, which were used in the variability analysis described below. The device continuously recorded dynamic LLT data throughout this period, providing a real-time graph that fluctuated according to changes in the lipid layer over time. The collected data were then analyzed to classify LLT variability into two categories, high- (HV) and low-variability (LV), based on the overall shape of the LLT graph. Classification was performed by visually inspecting the appearance of a dynamic graph (Fig 1). HV was defined as meeting any of the following criteria: (1) LLT does not remain within a 25 nm range for at least 10 s; (2) the SD of LLT exceeds 10 nm; (3) the slope of the LLT graph illustrates abrupt changes, specifically when the slope transitions from positive to negative or from negative to positive; and (4) the difference between the maximum and minimum LLT values exceeds 25 nm. Patients who did not meet any of these criteria were classified as having LV. Two ophthalmologists who were blinded to each other’s assessments independently classified LLT variability according to predefined criteria. Numeric parameters obtained from the LipiView® II included the SD of LLT and the number of blinks within a 20-s period to estimate the blinking frequency (BF) per minute.

### Statistical analysis

Statistical analyses were conducted using SPSS software (version 23.0; SPSS Inc., Chicago, IL, USA). The normality of all parameters was initially assessed using the Kolmogorov–Smirnov test. The comparison between the HV and LV groups in patients with device-reported high-range LLT was assessed using generalized estimating equations (GEE) with a Gaussian family for continuous variables, including age, DED, and MGD parameters. For categorical variables, sex distribution, and prevalence of medical conditions, GEE with a binomial family was used. When data from both eyes of a patient were available, both eyes were included, and the eye was the unit of analysis. Because both eyes of some patients were included, all comparisons were performed using GEE with an independence working correlation structure and robust standard errors, treating each patient as a cluster, to account for inter-eye correlation. Multivariable GEE logistic regression analysis was performed to identify factors associated with HV. ORs with 95% CIs were calculated. The final dataset included 85 eyes from 62 patients with concordant LLT variability classification. Complete data were available for all variables except the SD of LLT and blinking frequency, which were missing in one eye. Descriptive analyses used all available data, and GEE logistic regression was performed using complete cases for the variables included in the model. A *p*-value of <0.05 was considered statistically significant for all analyses.

## Results

### Demographics of the study population

Eighty-seven eyes were assessed. Two ophthalmologists agreed on LLT variability classification for 85 eyes (97.7%). Among the concordant cases, 27 eyes (31.8%) were classified as LV and 58 eyes (68.2%) as HV. Two eyes (2.2%) with discordant classifications were excluded from subsequent analyses. Inter-rater reliability was excellent, with Cohen’s κ = 0.95. Therefore, all subsequent analyses were performed on the 85 eyes (from 62 patients) with a concordant classification. The demographic characteristics of the study participants are summarized in Table 1.

**Table 1:** Demographic and clinical characteristics of the study cohort with device-reported high-range mean LLT (≥ 100 nm / “100+”).

Patient-level data	Total (n = 62)
**Sex**	
**Male (n, %)**	20 (32.3)
**Female (n, %)**	42 (67.7)
**Age (years)**	70.8 ± 10.7
**HTN (n, %)**	31 (50.0)
**DM (n, %)**	20 (32.3)
**Dyslipidemia (n, %)**	29 (46.8)
**Eye-level data**	**Total (n = 85)**
**LLT variability**	
**HV (n, %)**	58 (68.2)
**LV (n, %)**	27 (31.8)
**TBUT (s)**	5.40 ± 5.17
**Corneal Stain (0–5)**	1.92 ± 1.58
**Conjunctival Stain (0–10)**	4.71 ± 3.31
**MQ (0–24)**	11.74 ± 6.02
**ME (0–8)**	3.40 ± 2.40
**SPEED score (0–28)**	3.67 ± 4.17
**BF (/min)**	15.74 ± 14.84
**SD of LLT**	8.08 ± 5.30
**Meiboscore (0–6)**	2.39 ± 1.48

BF, blinking frequency; DM, diabetes mellitus; HTN, hypertension; HV, high-variability; LLT, lipid layer thickness; LV, low-variability; ME, meibum expressibility; MQ, meibum quality; SD, standard deviation; SPEED, standard patient evaluation of eye dryness; TBUT, tear break-up time.

BF and SD of LLT were calculated from 84 eyes because these values were unavailable for one eye.

Demographic characteristics are reported at the patient level (n = 62), whereas the LLT variability classification and all clinical ocular parameters are reported at the eye level (n = 85).

### Impact of LLT variability on DED and MGD

**Fig 2:**
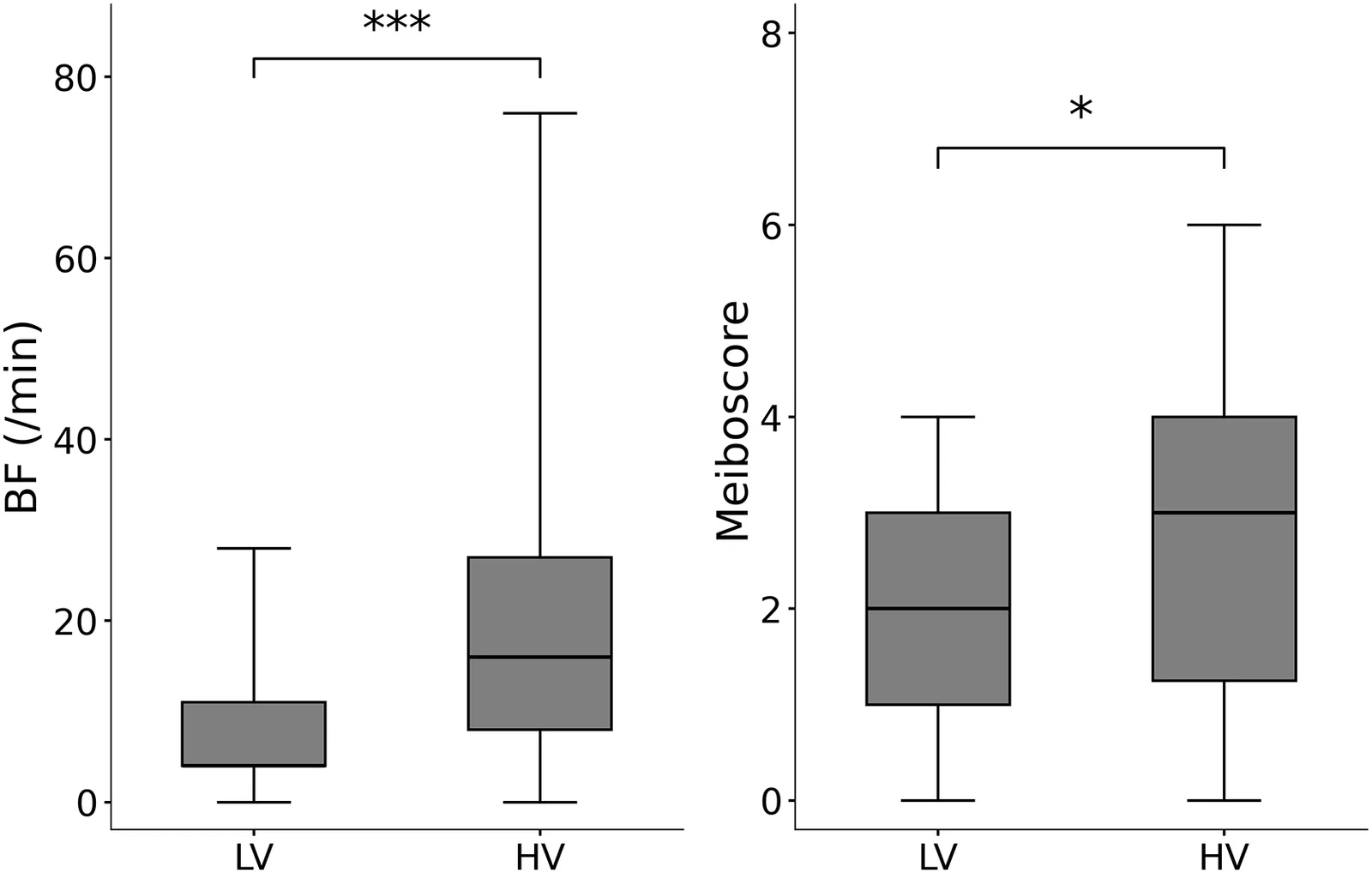
Box plots of blinking frequency and meiboscore by lipid layer thickness variability. Box plots comparing (A) blinking frequency (BF) and (B) meiboscore between high- (HV) and low-variability (LV) lipid layer thickness (LLT) pattern groups. The left box in each plot represents the LV group, and the right box represents the HV group. The center lines indicate medians, boxes indicate interquartile ranges, and whiskers indicate minimum and maximum values. HV eyes exhibited increased BF and higher median meiboscores than LV eyes. Statistical significance was assessed using generalized estimating equations: ***p < 0.001, *p < 0.05.

GEEs were employed to compare the mean values of various DED and MGD parameters between the HV and LV groups (Table 2). Although the SD of LLT was used to define the HV group, Table 2 illustrates and compares the LLT variability between the LV and HV LLT groups. Notably, the HV group exhibited remarkably higher meiboscores (2.62 ± 1.53) compared to the LV group (1.89 ± 1.25, *p* = 0.042), indicating greater MG dropout compared to the LV group. Additionally, the HV group depicted elevated SPEED scores (4.52 ± 4.65), and BF (19.41 ± 15.85) compared to the LV pattern group (1.83 ± 1.98 and 7.54 ± 7.53, respectively); the statistical significance of both parameters was *p* < 0.001. Corneal and conjunctival staining scores were numerically higher in the HV group, but these differences did not reach statistical significance. MQ and ME did not differ significantly between the two groups. These group differences in BF and meiboscore are further illustrated in Fig 2, which shows higher median BF and meiboscore values in the HV group than in the LV group.

**Table 2:** Comparison of clinical parameters between high- and low-variability lipid layer thickness pattern groups.

	HV (n = 58)	LV (n = 27)	*p*
**Age (years)**	70.09 ± 12.12	70.56 ± 12.65	0.902^a^
**Sex**			0.609^b^
**Male (n)**	18	10	
**Female (n)**	40	17	
**HTN (N)**	29	14	0.885^b^
**DM (N)**	20	10	0.835^b^
**Dyslipidemia (n)**	23	13	0.493^b^
**TBUT (s)**	4.80 ± 4.17	6.68 ± 6.78	0.237^a^
**Corneal Stain (0–5)**	2.16 ± 1.55	1.41 ± 1.55	0.053^a^
**Conjunctival Stain (0–10)**	5.19 ± 3.30	3.67 ± 3.15	0.059^a^
**MQ (0–24)**	12.53 ± 6.45	10.04 ± 4.63	0.091^a^
**ME (0–8)**	3.36 ± 2.42	3.48 ± 2.41	0.851^a^
**SPEED score (0–28)**	4.52 ± 4.65	1.85 ± 1.98	0.001^a^*
**BF (/min)**	19.41 ± 15.85	7.54 ± 7.53	<0.001^a^*
**SD of LLT**	9.29 ± 5.57	5.38 ± 3.40	<0.001^a^*
**Meiboscore (0–6)**	2.61 ± 1.53	1.89 ± 1.25	0.042^a^*

^a^Generalized estimating equations (GEE) with a Gaussian family.

^b^GEE with a binomial family.

*Statistically significant (*p* < 0.05).

The eye was the unit of analysis; age, sex, and systemic comorbidities (HTN, DM, dyslipidemia) are patient-level characteristics presented at the eye level.

BF, blinking frequency; DM, diabetes mellitus; HTN, hypertension; HV, high-variability; LLT, lipid layer thickness; LV, low-variability; ME, meibum expressibility; MQ, meibum quality; SD, standard deviation; SPEED, standard patient evaluation of eye dryness; TBUT, tear break-up time.

Logistic regression analysis was also conducted to evaluate the factors associated with an HV LLT pattern, controlling for various parameters including age, sex, systemic disease, and DED/MGD parameters (Table 3). Similar trends were confirmed in the multivariate analysis: patients with an elevated meiboscore had significantly higher odds of exhibiting an HV LLT pattern (odds ratio [OR] = 1.666, 95% confidence interval [CI], 1.069–2.597, *p* = 0.024). Additionally, elevated SPEED scores (OR = 1.306, 95% CI: 1.073–1.589, *p* = 0.008) and increased BF (OR = 1.148, 95% CI: 1.047–1.259, *p* = 0.003) were also associated with an HV LLT pattern. Overall, these logistic regression findings underscore that, even after adjustment for demographic and clinical variables, a greater MG dropout, more severe DED-related findings, higher SPEED score, and increased BF remained associated with an HV LLT pattern.

**Table 3:** Logistic regression analysis of factors associated with high-variability lipid layer thickness patterns.

	OR	95% CI	*p*
**Sex**	0.303	0.062–1.483	0.141
**Age**	1.015	0.939–1.096	0.712
**HTN**	0.734	0.144–3.752	0.711
**DM**	1.446	0.374–5.587	0.593
**Dyslipidemia**	0.986	0.224–4.327	0.985
**TBUT (s)**	1.084	0.950–1.236	0.230
**Corneal Stain (0–5)**	1.249	0.707–2.206	0.444
**Conjunctival Stain (0–10)**	1.124	0.871–1.450	0.368
**MQ (0–24)**	1.030	0.902–1.175	0.665
**ME (0–8)**	1.009	0.709–1.436	0.961
**SPEED score (0–28)**	1.306	1.073–1.589	0.008*
**BF (/min)**	1.148	1.047–1.259	0.003*
**Meiboscore (0–6)**	1.666	1.069–2.597	0.024*

*Statistically significant (*p* < 0.05) according to logistic regression analysis.

BF, blinking frequency; CI, confidence interval; DM, diabetes mellitus; HTN, hypertension; ME, meibum expressibility; MQ, meibum quality; OR, odds ratio; SPEED, standard patient evaluation of eye dryness; TBUT, tear break-up time.

For sex, the odds ratio represents female sex relative to male sex.

## Discussion

In this retrospective study, eyes with device-reported high-range LLT values (≥ 100 nm or “100+” by LipiView® II) were stratified into HV and LV pattern groups based on dynamic LLT trace variability. Because LipiView® II values in this range should not be interpreted as calibrated absolute LLT, the present analysis focused on qualitative temporal variability rather than the physiologic sufficiency of a specific LLT cutoff. Despite no significant differences in MQ and ME, the HV LLT pattern group exhibited significantly higher meiboscores and SPEED scores than the LV group, with a trend toward higher corneal and conjunctival staining scores. This suggests that dynamic LLT trace variability may provide complementary information about meibomian gland morphology and ocular surface status beyond the average device-reported LLT value. This statistical significance indicates that anatomical and morphological alterations in the MGs were more pronounced in the HV LLT group, even though MG function, as represented by MQ and ME, did not differ significantly between the HV and LV groups. Furthermore, patients in this group experienced substantially more severe subjective DED symptoms, as reflected by elevated SPEED scores, than those with an LV LLT pattern. These observations suggest that, although the device-reported mean LLT was in the high range, the HV LLT pattern is associated not only with increased MG dropout but also with a trend toward greater ocular surface damage, as suggested by numerically higher corneal and conjunctival scores, implying that the ocular surface instability observed in MGD may stem from underlying morphological changes in the MGs.

Logistic regression analysis further corroborated these findings. After controlling for confounding variables, regression analysis demonstrated that the meiboscore remained associated with higher odds of exhibiting an HV LLT pattern after adjustment, reinforcing the idea that dynamic LLT variability reflects MG dropout beyond MQ and ME. Other factors, including SPEED and BF, also exhibited positive associations with the HV, further supporting their contributory roles in tear film instability. These multivariate findings echo the trends observed in the univariate analysis, underscoring that, even in the presence of device-reported high-range mean LLT values and similar MQ and ME, the HV LLT pattern is robustly associated with both anatomical alterations in the MGs and increased severity of DED symptoms.

Traditional evaluation methods relying solely on numerical LLT values may overlook critical functional information. LipiView® II is an instrument used to measure the LLT of the tear film, where one ICU approximately corresponds to 1 nm of LLT. LLT values exceeding 100 nm are often recorded as “100+,” and the accompanying LLT graph patterns frequently fall into a gray zone, limiting their interpretability. These limitations underscore the importance of qualitative evaluation of LLT patterns.

This study supports the notion that the qualitative evaluation of LLT patterns offers critical insights that numerical measurements alone may not capture. The significant association between the HV LLT pattern and increased MG dropout, as well as more severe DED symptoms, together with a trend toward greater ocular surface staining, suggests that the dynamic LLT graph may represent a candidate marker for early or subclinical MG pathology that warrants prospective evaluation. The observed HV LLT pattern may reflect subtle anatomical alterations within the MGs that disrupt uniform lipid secretion, ultimately leading to tear film instability.

One possible hypothetical mechanism, without a direct causal relationship, is that even when MG dropout occurs, the remaining glands may compensate by increasing secretion, resulting in a device-reported high-range mean LLT and no changes in ME or MQ. However, this compensatory secretion may not achieve uniform lipid layer distribution, causing intermittent or localized thinning of the LLT. Each focal thinning can compromise tear film stability, leading to ocular surface staining and subclinical epithelial damage. Taken together, the higher SPEED scores and numerically greater ocular surface staining in the HV group may indicate greater ocular surface discomfort or epithelial compromise, which could partly explain the increased BF observed in these eyes. Cohen, et al. [22] demonstrated that high-resolution LLT mapping in patients with lipid-deficient DED revealed a markedly non-uniform lipid layer, in contrast to the uniform distribution seen in healthy controls. Consequently, the non-uniform distribution of the lipid layer can elicit increased discomfort, which in turn may prompt a compensatory elevation in the BF to reestablish lipid coverage. Ultimately, this hypothetical cascade suggests that a seemingly thick mean LLT and preserved MG function do not preclude local tear film deficiencies and that pattern instability, ocular surface damage, and compensatory BF should be evaluated even when average LLT metrics appear unremarkable.

These findings suggest that MG dropout itself may be associated with increased LLT variability, even in eyes with device-reported high-range mean LLT values, regardless of MQ or ME. An HV LLT pattern could serve as a possible early indicator of MGD progression, emerging before significant changes in other parameters, such as ME or MQ. Consequently, in patients with DED symptoms but whose mean LLT and MG functions appear normal, clinicians should still evaluate LLT variability, blink dynamics, and the potential for MG dropout rather than relying solely on averaged metrics.

This study has several limitations that warrant consideration. The retrospective design and visual pattern classification of this study may limit causal inference and introduce observer bias. In addition, because both eyes of some patients were included, the observations were not fully independent; however, a GEE accounting for within-patient correlation confirmed that the principal associations remained significant. The modest sample size and predominantly middle-aged cohort may restrict generalizability. An important limitation concerns the measurement principle of the LipiView® II itself. Interferometric estimation of LLT based on color analysis has a narrow unambiguous range [23] and depends on assumptions within a proprietary fitting algorithm, which is not disclosed by the manufacturer and could not be independently calibrated against films of known thickness in this retrospective study. The tear film lipid layer is also a non-uniform, multilayered structure whose thickness varies spatially and temporally [24,25]. Therefore, absolute values reported as “100+” nm should be interpreted with caution. Although the absolute calibration accuracy of the device could not be verified, previous work has examined the repeatability of LipiView® interferometric LLT measurement [26]. Because our analysis relies on relative temporal changes within a single continuous acquisition rather than on absolute LLT values, measurement reproducibility was considered more relevant than absolute calibration accuracy for interpreting the temporal variability observed within each eye. For these reasons, we did not rely on absolute LLT values but instead adopted a qualitative classification of the temporal variability pattern, which is expected to be more robust to absolute calibration error because it reflects relative changes recorded within a single acquisition under identical optical conditions. Nonetheless, two of the four classification criteria incorporate fixed nanometer thresholds, and the SD of LLT also remains device-derived. Therefore, these variability parameters should not be regarded as fully independent of the device scale. However, the concordance among visual trace morphology, SD differences, and clinical associations supports the internal consistency of the classification. In addition, TBUT was measured after instillation of a preservative-containing topical anesthetic rather than non-preserved saline, which is not standard practice and may have affected absolute TBUT values. However, because the same agent was applied uniformly to all participants, its influence on between-group comparisons is expected to be limited. Therefore, prospective large-scale studies across diverse populations are required. Longitudinal analyses will clarify how LLT patterns evolve over time and respond to treatment. Integrating high-resolution LLT mapping and more quantitative surface-analysis techniques, such as ellipsometry, with automated analytics can further refine our understanding of tear film lipid dynamics and MG pathology.

Summarily, numerical LLT measurements alone may mask the MG pathology. This study suggests that even in patients with DED symptoms who have a device-reported high-range mean lipid layer, LLT pattern variability can be qualitatively evaluated. When LLT variability is elevated, adjunctive assessment of the meiboscore and blinking patterns may complement existing assessments of MGD.

## Supporting information

S1 Data Minimal anonymized dataset. De-identified data for all 85 eyes, including the variables used in Tables 1–3 and the high-/low-variability classification.
(XLSX)
