# Supplementary material for: Qualitative analysis and clinical implications of lipid layer thickness variability: A retrospective, cross-sectional study
Source: PLoS One. 2026 Sep 15;21(9):e0357020. doi: 10.1371/journal.pone.0357020 (PMC13577391; doi:10.1371/journal.pone.0357020)
Supplement: Peer review history

# Peer review: Decision Letter 0

**DOI:** 10.1371/journal.pone.0357020.r001

**Authors:** Nick Fogt

26 May 2026

Dear Dr. Na,

Thank you for submitting your manuscript to PLOS ONE. After careful consideration, we feel that it has merit but does not fully meet PLOS ONE’s publication criteria as it currently stands. Therefore, we invite you to submit a revised version of the manuscript that addresses the points raised during the review process.

Please submit your revised manuscript by Jul 10 2026 11:59PM. If you will need more time than this to complete your revisions, please reply to this message or contact the journal office at plosone@plos.org. When you're ready to submit your revision, log on to https://www.editorialmanager.com/pone/ and select the 'Submissions Needing Revision' folder to locate your manuscript file.

- A letter that responds to each point raised by the academic editor and reviewer(s). You should upload this letter as a separate file labeled 'Response to Reviewers'.
- A marked-up copy of your manuscript that highlights changes made to the original version. You should upload this as a separate file labeled 'Revised Manuscript with Track Changes'.
- An unmarked version of your revised paper without tracked changes. You should upload this as a separate file labeled 'Manuscript'.

If you would like to make changes to your financial disclosure, please include your updated statement in your cover letter. Guidelines for resubmitting your figure files are available below the reviewer comments at the end of this letter.

If applicable, we recommend that you deposit your laboratory protocols in protocols.io to enhance the reproducibility of your results. Protocols.io assigns your protocol its own identifier (DOI) so that it can be cited independently in the future. For instructions see: https://journals.plos.org/plosone/s/submission-guidelines#loc-laboratory-protocols. Additionally, PLOS ONE offers an option for publishing peer-reviewed Lab Protocol articles, which describe protocols hosted on protocols.io. Read more information on sharing protocols at https://plos.org/protocols?utm_medium=editorial-email&utm_source=authorletters&utm_campaign=protocols.

As the corresponding author, your ORCID iD is verified in the submission system and will appear in the published article. PLOS supports the use of ORCID, and we encourage all coauthors to register for an ORCID iD and use it as well. Please encourage your coauthors to verify their ORCID iD within the submission system before final acceptance, as unverified ORCID iDs will not appear in the published article. *Only*  the individual author can complete the verification step; PLOS staff *cannot*  verify ORCID iDs on behalf of authors.

We look forward to receiving your revised manuscript.

Kind regards,

Nick Fogt

Academic Editor

PLOS One

Journal Requirements:

When submitting your revision, we need you to address these additional requirements.

1. Please ensure that your manuscript meets PLOS ONE's style requirements, including those for file naming. The PLOS ONE style templates can be found at

https://journals.plos.org/plosone/s/file?id=wjVg/PLOSOne_formatting_sample_main_body.pdf and

https://journals.plos.org/plosone/s/file?id=ba62/PLOSOne_formatting_sample_title_authors_affiliations.pdf

2. We note that you have indicated that there are restrictions to data sharing for this study. PLOS only allows data to be available upon request if there are legal or ethical restrictions on sharing data publicly. For more information on unacceptable data access restrictions, please see http://journals.plos.org/plosone/s/data-availability#loc-unacceptable-data-access-restrictions.

Before we proceed with your manuscript, please address the following prompts:

a) If there are ethical or legal restrictions on sharing a de-identified data set, please explain them in detail (e.g., data contain potentially identifying or sensitive patient information, data are owned by a third-party organization, etc.) and who has imposed them (e.g., a Research Ethics Committee or Institutional Review Board, etc.). Please also provide contact information for a data access committee, ethics committee, or other institutional body to which data requests may be sent.

b) If there are no restrictions, please upload the minimal anonymized data set necessary to replicate your study findings to a stable, public repository and provide us with the relevant URLs, DOIs, or accession numbers. For a list of recommended repositories, please see

https://journals.plos.org/plosone/s/recommended-repositories. You also have the option of uploading the data as Supporting Information files, but we would recommend depositing data directly to a data repository if possible.

We will update your Data Availability statement on your behalf to reflect the information you provide.

3. If the reviewer comments include a recommendation to cite specific previously published works, please review and evaluate these publications to determine whether they are relevant and should be cited. There is no requirement to cite these works unless the editor has indicated otherwise.

Additional Editor Comments:

The manuscript has been reviewed by two reviewers. Reviewer #1 has pointed out (and included references) that there are limitations of the instrumentation that must be addressed. Please acknowledge these limitations and indicate whether and how these limitations could affect the study results. Reviewer #2 has asked for a number of clarifications in the cited references, and has asked for clarification with regard to some of the methodology and the results (especially the interpretation of Figures 1a and 1b). Please thoroughly address each comment.

[Note: HTML markup is below. Please do not edit.]

Reviewers' comments:

Reviewer's Responses to Questions

**Comments to the Author**

1. Is the manuscript technically sound, and do the data support the conclusions?

Reviewer #1: No

Reviewer #2: Yes

**********

2. Has the statistical analysis been performed appropriately and rigorously?

Reviewer #1: No

Reviewer #2: Yes

**********

3. Have the authors made all data underlying the findings in their manuscript fully available?

The PLOS Data policy

Reviewer #1: No

Reviewer #2: Yes

**********

4. Is the manuscript presented in an intelligible fashion and written in standard English?

Reviewer #1: Yes

Reviewer #2: Yes

**********

Reviewer #1: The results of this study are based on information provided by an interferometer. The LipiViewII has an extremely limited range that may be accurate from 20 to below 100 nm. The shortcoming of interferometry for lipid layer measurements has been previously described a long time ago; Kitagawa K Thin-film thickness profile measurement by three-wavelength interference color analysis. Appl Opt 2013;52:1998. doi: 10.1364/AO.52.001998. and McDonald JE. Surface phenomena of tear films. Trans Am Ophthalmol Soc 1968;66:905–39 and ,more recently Glasgow BJ Ocul Surf. 2019 Feb 25;17(2):341–346. doi: 10.1016/j.jtos.2019.02.008. Therefore any measurement over 100 nm is not in the range of the instrument and those under that range are subject to the limitations of color analysis. The instrument software has to make an assumption of thickness to generate a data thickness and the variation in this estimate is usually very large. The fitting algorithm should be included in the methods. The authors could calibrate their instrument's accuracy with films of known thickness but this was not done. Since tears in vitro have been shown to have thicknesses that are well outside the range of this clinical instrument, and because the tear film thickness in vitro encompasses ranges that vary from region to region and day to day in tears from subjects without dry eye, the results and conclusions are suspect. The authors are not at fault here, it is the limitation of the technology.

Reviewer #2: General Comments

1) The authors should be cautious when stating that 100 nm LLT is “considered sufficient to maintain tear film stability.” This is not exactly correct. The referenced paper (#13) even states that patients who had a LLT of 100 STILL had fast tear film break up times/tear film stability which is contrary to the authors statement. The authors also claim this in the abstract (line 27). This is also incorrect. Please rephrase these sentences and do a deeper search of the literature for appropriate LLT cutoffs if the authors want to report these values.

a. The literature varies widely, anywhere from 60 nm to 80 nm

2) The last several lines of the introduction (85-89) go into the results of the study which should not be a part of the introduction

3) Since this was a retrospective study, how are the authors certain that there were 5 minute intervals between the testing done retrospectively? (Lines 127-129)

4) The method used for evaluating tear film break up time is not standard. The clinician used proparaine to wet the fluorescein strips, which is inherently preserved with BAK which can disrupt the tears. Why wasn’t saline used to wet the fluorescein strips, as is standard practice?

5) In the description for classifying high variability LLT- the authors now mention min and max LLT ; the authors should clarify earlier that the Lipiview also reports this data otherwise the reader may be confused where these values come from.

6) In Figure 1a, the example with low-variablity – does it seem to show that the variability did not change because the patient did not blink? Figure 1b shows that the patient blinked at least twice.

Specific Comments

1) Line 50 – what is meant by “generalized” condition?

2) Please add to the description of the Lipiview the area over which the tear film and LLT is measured.

3) Please also clarify line 66-the device provides an average LLT over time or over a certain area?

4) The lipiview also provides a minimum and maximum LLT.

5) Line 104- clarify what is meant by the assessment of “MG” – structure? Function?

6) Table 2 – dyslipidemia, LV – the number 13 has an S following it that should be deleted

**********

what does this mean?). If published, this will include your full peer review and any attached files.

If you choose “no”, your identity will remain anonymous but your review may still be made public.

**Do you want your identity to be public for this peer review?**  For information about this choice, including consent withdrawal, please see our Privacy Policy

Reviewer #1: No

Reviewer #2: No

**********

[NOTE: If reviewer comments were submitted as an attachment file, they will be attached to this email and accessible via the submission site. Please log into your account, locate the manuscript record, and check for the action link "View Attachments". If this link does not appear, there are no attachment files.]

To ensure your figures meet our technical requirements, please review our figure guidelines: https://journals.plos.org/plosone/s/figures

You may also use PLOS’s free figure tool, NAAS, to help you prepare publication quality figures: https://journals.plos.org/plosone/s/figures#loc-tools-for-figure-preparation.

NAAS will assess whether your figures meet our technical requirements by comparing each figure against our figure specifications.

# Author comment: Author response to Decision Letter 1

**DOI:** 10.1371/journal.pone.0357020.r002

6 Jul 2026

Response to Reviewers

Manuscript ID: PONE-D-25-47863

Title: Qualitative analysis and clinical implications of lipid layer thickness variability: a retrospective, cross-sectional study

Dear Dr. Nick Fogt and Reviewers,

We would like to express our sincere gratitude to the Academic Editor and Reviewers for dedicating your time to review our manuscript (PONE-D-25-47863). We deeply appreciate your constructive criticisms and insightful suggestions. We completely agree with your valuable points, which have undoubtedly helped us improve the clarity and quality of our manuscript.

We have carefully revised the manuscript according to your recommendations. Our point-by-point responses to the reviewers' comments are detailed below. Changes are highlighted in the marked-up manuscript, and reference numbers refer to the revised reference list.

Journal Requirements:

When submitting your revision, we need you to address these additional requirements.

1. Please ensure that your manuscript meets PLOS ONE's style requirements, including those for file naming. The PLOS ONE style templates can be found at

https://journals.plos.org/plosone/s/file?id=wjVg/PLOSOne_formatting_sample_main_body.pdf and

https://journals.plos.org/plosone/s/file?id=ba62/PLOSOne_formatting_sample_title_authors_affiliations.pdf

Response: Thank you for this reminder. We have carefully reviewed our manuscript in accordance with the PLOS ONE style requirements, including the file-naming conventions and formatting guidance provided in the title/authors/affiliations and main-body sample templates. We confirm that the manuscript conforms to these requirements. We appreciate the editor’s comment, which allowed us to verify the formatting once again.

2. We note that you have indicated that there are restrictions to data sharing for this study. PLOS only allows data to be available upon request if there are legal or ethical restrictions on sharing data publicly. For more information on unacceptable data access restrictions, please see http://journals.plos.org/plosone/s/data-availability#loc-unacceptable-data-access-restrictions.

Before we proceed with your manuscript, please address the following prompts:

a) If there are ethical or legal restrictions on sharing a de-identified data set, please explain them in detail (e.g., data contain potentially identifying or sensitive patient information, data are owned by a third-party organization, etc.) and who has imposed them (e.g., a Research Ethics Committee or Institutional Review Board, etc.). Please also provide contact information for a data access committee, ethics committee, or other institutional body to which data requests may be sent.

b) If there are no restrictions, please upload the minimal anonymized data set necessary to replicate your study findings to a stable, public repository and provide us with the relevant URLs, DOIs, or accession numbers. For a list of recommended repositories, please see

https://journals.plos.org/plosone/s/recommended-repositories. You also have the option of uploading the data as Supporting Information files, but we would recommend depositing data directly to a data repository if possible.

We will update your Data Availability statement on your behalf to reflect the information you provide.

Response: We are grateful for this valuable suggestion. We confirm that there are no legal or ethical restrictions on sharing the de-identified data. We have prepared the minimal anonymized data set required to replicate our findings and provide it as a Supporting Information file (S1 Data).

In addition, during preparation of this data set, we re-verified and refined the data; the final data set comprises 85 eyes from 62 patients, and the principal findings were unchanged. Because both eyes of some patients were included, the eye was treated as the unit of analysis, and we additionally performed a generalized estimating equation analysis clustered by patient, which confirmed the robustness of our findings to within-patient correlation. These points are now described in the Methods and Results.

3. If the reviewer comments include a recommendation to cite specific previously published works, please review and evaluate these publications to determine whether they are relevant and should be cited. There is no requirement to cite these works unless the editor has indicated otherwise.

Response: We thank the editor for this guidance. Reviewer #1 recommended three publications regarding the limitations of interferometry for lipid layer measurement (Kitagawa, Appl Opt 2013; McDonald, Trans Am Ophthalmol Soc 1968; and Glasgow, Ocul Surf 2019). We reviewed and evaluated each of these works, found them to be directly relevant to the instrumentation limitations discussed in our manuscript, and have cited them accordingly (References 23–25). In addition, in response to Reviewer #2’s comments on appropriate LLT cutoffs, we identified and cited the primary sources for these values (Blackie, Cornea 2009 [Reference 13]; and Finis, Cornea 2013 [Reference 14]). We also added a study on the repeatability of LipiView® interferometric measurements (Zhao et al., BMC Ophthalmol 2015 [Reference 26]). We found all of these works to be relevant and have incorporated them into the revised manuscript.

Additional Editor Comments:

The manuscript has been reviewed by two reviewers. Reviewer #1 has pointed out (and included references) that there are limitations of the instrumentation that must be addressed. Please acknowledge these limitations and indicate whether and how these limitations could affect the study results. Reviewer #2 has asked for a number of clarifications in the cited references, and has asked for clarification with regard to some of the methodology and the results (especially the interpretation of Figures 1a and 1b). Please thoroughly address each comment.

Response: We thank the Academic Editor for these directions. We have acknowledged the instrumentation limitations raised by Reviewer #1 in both the Methods and the Discussion and explicitly indicated how they could affect the results and how our approach mitigates them (see our detailed reply to Reviewer #1, points 1–6). In brief, the LipiView® II output is now described as a device-derived interferometric estimate rather than a calibrated absolute thickness. We disclose that the fitting algorithm is proprietary, note that the device was not calibrated against films of known thickness, and explain that—because our analysis relies on the temporal variability patterns and on outcomes measured independently of the interferometric scale—the principal findings are robust to this limitation. We have also addressed each clarification requested by Reviewer #2, including the cited references, methodology, and interpretation of Figures 1a and 1b. Specifically, we clarified that variability is assessed based on the overall trace shape, independent of the number of blinks. A point-by-point response follows below.

[Reviewer #1]

The results of this study are based on information provided by an interferometer. The LipiViewII has an extremely limited range that may be accurate from 20 to below 100 nm. The shortcoming of interferometry for lipid layer measurements has been previously described a long time ago; Kitagawa K Thin-film thickness profile measurement by three-wavelength interference color analysis. Appl Opt 2013;52:1998. doi: 10.1364/AO.52.001998. and McDonald JE. Surface phenomena of tear films. Trans Am Ophthalmol Soc 1968;66:905–39 and ,more recently Glasgow BJ Ocul Surf. 2019 Feb 25;17(2):341–346. doi: 10.1016/j.jtos.2019.02.008. Therefore any measurement over 100 nm is not in the range of the instrument and those under that range are subject to the limitations of color analysis. The instrument software has to make an assumption of thickness to generate a data thickness and the variation in this estimate is usually very large. The fitting algorithm should be included in the methods. The authors could calibrate their instrument's accuracy with films of known thickness but this was not done. Since tears in vitro have been shown to have thicknesses that are well outside the range of this clinical instrument, and because the tear film thickness in vitro encompasses ranges that vary from region to region and day to day in tears from subjects without dry eye, the results and conclusions are suspect. The authors are not at fault here, it is the limitation of the technology.

Response: We are grateful for this important comment and fully acknowledge the limitations of the instrument. In response, we have revised the manuscript to ensure that our conclusions do not depend on the absolute accuracy of the interferometric reading. Specifically:

1. Measurement principle and fitting algorithm (Methods). We have clarified the description of the measurement and no longer state that the device “quantifies” thickness. The Methods section now reads:

“The mean LLT was measured using a LipiView® II interferometer, which provides device-derived interferometric estimates of TFLL, reported in nanometers (nm) or interferometric color units (ICUs). The conversion from the recorded interference pattern to a thickness value relies on the manufacturer’s proprietary fitting algorithm, which is not publicly disclosed.”

We have explicitly noted that the fitting algorithm is proprietary and not released by the manufacturer. Therefore, it cannot be independently reproduced or fully described.

2. Acknowledgment of instrumentation limitations (Limitations), citing the recommended works:

“Interferometric estimation of LLT based on color analysis has a narrow unambiguous range and depends on assumptions within a proprietary fitting algorithm, which is not disclosed by the manufacturer and could not be independently calibrated against films of known thickness in this retrospective study [23]. The tear film lipid layer is also a non-uniform, multilayered structure whose thickness varies spatially and temporally [24,25]. Therefore, absolute values reported as “100+” nm should be interpreted with caution.”

3. Impact of this limitation and mitigation approach:

Because absolute values in this range are unreliable, we did not rely on absolute thickness. Instead, we adopted a qualitative classification of the temporal variability pattern, which reflects relative changes recorded within a single acquisition under identical optical conditions. We have also tempered our previous wording and now state that the classification is not fully independent of the device scale:

“Nonetheless, two of the four classification criteria incorporate fixed nanometer thresholds, and the SD of LLT also remains device-derived. Therefore, these variability parameters should not be regarded as fully independent of the device scale. However, the concordance among visual trace morphology, SD differences, and clinical associations supports the internal consistency of the classification.”

4. The principal findings do not depend on the absolute LLT value. The clinical outcome measures associated with the variability pattern—meiboscore, SPEED score, corneal and conjunctival staining, and blinking frequency—were obtained independently of the interferometric LLT scale. In the revised analysis, between-group comparisons were performed using GEE models with robust standard errors, treating each patient as a cluster to account for within-patient inter-eye correlation. In addition, because inclusion required a device-reported LLT of ≥100 nm (“100+”), the mean LLT was effectively at the device ceiling and was not used as a graded predictor. Therefore, the observed between-group differences are driven by temporal variability patterns rather than by the absolute magnitude of the device-reported mean LLT.

5. Reproducibility versus absolute accuracy (Limitations). We added:

“Although the absolute calibration accuracy of the device could not be verified, previous work has examined the repeatability of LipiView® interferometric LLT measurements [26]. Because our analysis relies on relative temporal changes within a single continuous acquisition rather than on absolute LLT values, measurement reproducibility was considered more relevant to the within-eye comparisons reported here.”

6. Conclusions reframed as hypothesis-generating. We have tempered our conclusions so that they propose a candidate pattern biomarker that warrants prospective evaluation rather than an established diagnostic criterion—claims that do not require absolute-thickness accuracy. For example:

“…the dynamic LLT graph may represent a candidate marker for early or subclinical MG pathology.”

“When LLT variability is elevated, adjunctive assessment of the meiboscore and blinking patterns may complement existing assessments of MGD and merits prospective validation before clinical adoption.”

We agree that the absolute accuracy of this clinical instrument cannot be established retrospectively. We hope that, with these revisions, the reviewer will find that the study’s conclusions are appropriately qualified and supported by measures that are robust to this limitation.

[Reviewer #2]

General Comments

1) The authors should be cautious when stating that 100 nm LLT is “considered sufficient to maintain tear film stability.” This is not exactly correct. The referenced paper (#13) even states that patients who had a LLT of 100 STILL had fast tear film break up times/tear film stability which is contrary to the authors statement. The authors also claim this in the abstract (line 27). This is also incorrect. Please rephrase these sentences and do a deeper search of the literature for appropriate LLT cutoffs if the authors want to report these values.

a. The literature varies widely, anywhere from 60 nm to 80 nm

Response: We sincerely thank the reviewer for this insightful comment. We removed any implication that ≥100 nm is “sufficient” for stability throughout the Abstract, Introduction, and Discussion, and now describe ≥100 nm as a device-reported high-range or ceiling-level output rather than a calibrated absolute thickness. We have also added the relevant cutoff values with primary citations. The Introduction now reads:

“Although a thicker lipid layer is generally thought to confer greater resistance to evaporation, the reported LLT cutoffs associated with tear film stability vary widely across studies, ranging from approximately 60 to 80 nm [13,14], and a numerically high LLT does not necessarily indicate a stable tear film [15].”

Specifically, Blackie et al. (Reference 13) reported thin lipid layers (≤60 nm) in association with severe symptoms, while Finis et al (Reference 14) proposed a cutoff ≤75 nm for MGD; Reference 15 (Lee et al.) is retained to highlight that eyes with LLT values around 100 nm can still exhibit instability, consistent with the reviewer’s concern.

2) The last several lines of the introduction (85-89) go into the results of the study which should not be a part of the introduction

Response: We are grateful for this valuable suggestion, which has helped us improve the manuscript. We have removed the sentences summarizing the results from the end of the Introduction; these findings are retained in the Discussion and Conclusion

3) Since this was a retrospective study, how are the authors certain that there were 5 minute intervals between the testing done retrospectively? (Lines 127-129)

Response: Thank you for raising this important point. All examinations were performed according to our institution’s standardized dry eye examination protocol, which specifies a fixed test sequence with a minimum interval of 5 minutes between tests. This protocol is applied uniformly to all patients as part of routine clinical practice; thus, the testing sequence and minimum intervals are protocol-driven rather than determined on ad hoc basis. We have clarified this in the Methods section.

4) The method used for evaluating tear film break up time is not standard. The clinician used proparaine to wet the fluorescein strips, which is inherently preserved with BAK which can disrupt the tears. Why wasn’t saline used to wet the fluorescein strips, as is standard practice?

Response: We thank the reviewer for this important observation. At our institution, a

Attachment Submitted filename: Response_Letter.docx

# Editor report: Decision Letter 1

**DOI:** 10.1371/journal.pone.0357020.r003

**Authors:** Nick Fogt

12 Aug 2026

Qualitative analysis and clinical implications of lipid layer thickness variability: a retrospective, cross-sectional study

PONE-D-25-47863R1

Dear Dr. Na,

We’re pleased to inform you that your manuscript has been judged scientifically suitable for publication and will be formally accepted for publication once it meets all outstanding technical requirements.

Within one week, you’ll receive an e-mail detailing the required amendments. When these have been addressed, you’ll receive a formal acceptance letter and your manuscript will be scheduled for publication.

An invoice will be generated when your article is formally accepted. Please note, if your institution has a publishing partnership with PLOS and your article meets the relevant criteria, all or part of your publication costs will be covered. Please make sure your user information is up-to-date by logging into Editorial Manager at Editorial Manager® and clicking the ‘Update My Information' link at the top of the page. For questions related to billing, please contact billing support.

If your institution or institutions have a press office, please notify them about your upcoming paper to help maximize its impact. If they’ll be preparing press materials, please inform our press team as soon as possible -- no later than 48 hours after receiving the formal acceptance. Your manuscript will remain under strict press embargo until 2 pm Eastern Time on the date of publication. For more information, please contact onepress@plos.org.

Kind regards,

Nick Fogt

Academic Editor

PLOS One

Additional Editor Comments (optional):

Thank you for addressing the reviewer comments.

Reviewers' comments:

# Editor report: Acceptance letter

**DOI:** 10.1371/journal.pone.0357020.r004

**Authors:** Nick Fogt

PONE-D-25-47863R1

PLOS One

Dear Dr. Na,

I'm pleased to inform you that your manuscript has been deemed suitable for publication in PLOS One. Congratulations! Your manuscript is now being handed over to our production team.

At this stage, our production department will prepare your paper for publication. This includes ensuring the following:

* All references, tables, and figures are properly cited

* All relevant supporting information is included in the manuscript submission,

* There are no issues that prevent the paper from being properly typeset

You will receive further instructions from the production team, including instructions on how to review your proof when it is ready. Please keep in mind that we are working through a large volume of accepted articles, so please give us a few days to review your paper and let you know the next and final steps.

Lastly, if your institution or institutions have a press office, please let them know about your upcoming paper now to help maximize its impact. If they'll be preparing press materials, please inform our press team within the next 48 hours. Your manuscript will remain under strict press embargo until 2 pm Eastern Time on the date of publication. For more information, please contact onepress@plos.org.

You will receive an invoice from PLOS for your publication fee after your manuscript has reached the completed accept phase. If you receive an email requesting payment before acceptance or for any other service, this may be a phishing scheme. Learn how to identify phishing emails and protect your accounts at https://explore.plos.org/phishing.

If we can help with anything else, please email us at customercare@plos.org.

Thank you for submitting your work to PLOS One and supporting open access.

Kind regards,

PLOS ONE Editorial Office Staff

on behalf of

Dr. Nick Fogt

Academic Editor

PLOS One
